# Pluronic F127-Folate Coated Super Paramagenic Iron Oxide Nanoparticles as Contrast Agent for Cancer Diagnosis in Magnetic Resonance Imaging

**DOI:** 10.3390/polym11040743

**Published:** 2019-04-25

**Authors:** Hieu Vu-Quang, Mads Sloth Vinding, Thomas Nielsen, Marcus Görge Ullisch, Niels Chr. Nielsen, Dinh-Truong Nguyen, Jørgen Kjems

**Affiliations:** 1 NTT High-Tech Institute, Nguyen Tat Thanh University, Ho Chi Minh City 70000, Vietnam; 2 School of Biotechnology, Tan Tao University, Long An 82000, Vietnam; truong.nguyen@ttu.edu.vn; 3 Interdisciplinary Nanoscience Center (iNANO), Aarhus University, DK-8000 Aarhus, Denmark; msv@cfin.au.dk (M.S.V.); tn@ase.au.dk (T.N.); ullisch@inano.au.dk (M.G.U.); ncn@chem.au.dk (N.C.N.); jk@mbg.au.dk (J.K.); 4 Department of Molecular Biology, Aarhus University, DK-8000 Aarhus, Denmark

**Keywords:** SPION, contrast agent, MRI, cancer diagnosis, folate receptor, pluronic F127

## Abstract

Contrast agents have been widely used in medicine to enhance contrast in magnetic resonance imaging (MRI). Among them, super paramagnetic iron oxide nanoparticles (SPION) have been reported to have low risk in clinical use. In our study, F127-Folate coated SPION was fabricated in order to efficiently target tumors and provide imaging contrast in MRI. SPION alone have an average core size of 15 nm. After stabilizing with Pluronic F127, the nanoparticles reached a hydrodynamic size of 180 nm and dispersed well in various kinds of media. The F127-Folate coated SPION were shown to specifically target folate receptor expressing cancer cells by flow cytometry analysis, confocal laser scanning microscope, as well as in vitro MRI. Furthermore, in vivo MRI images have shown the enhanced negative contrast from the F127-Folate coated SPION in tumor-bearing mice. In conclusion, our F127-Folate coated SPION have shown great potential as a contrast agent in MRI, as well as in the combination with drug delivery for cancer therapy.

## 1. Introduction

Since the first discovery of the difference between cancer and normal tissue in the 1970′s by Raymond Vahan Damadian, a number of cancer diagnostic MRI methods have been developed [1]. Many inventions and ideas have been introduced to improve the imaging contrast, in order to achieve high diagnostic accuracy. One idea in particular, is the use of MRI contrast agents. There are two contrast agent types have been approved for use in medicine, including gadolinium based T1 contrast agents and super paramagnetic iron oxide nanoparticles (SPION) based T2 contrast agents [2,3,4]. Among them, SPION are more desirable due to their low toxicity when used [5]. Resovist and Feridex are two types of SPION that have recently been made available on the drug market. They have not only been approved for use in MRI of the liver, but have also entered clinical trials (phase II) for lymph node metastatic diagnosis [6]. However, these diagnoses are “indirect” as they rely on the position and migration of macrophages in the organs. Thus, it leads to difficulty in diagnosis when working with organs and tissues that have a small macrophage population, such as cancers. On the other hand, the ‘direct’ diagnosis, that is based on the accumulation of SPION in the tumor region, is preferable because it can provide the specific contrast in the images.

Immunological barriers play a crucial role in the distribution of nanoparticles. After parenteral injection, nanoparticles circulate in the system and are recognized by circulating monocytes and macrophages. They are then phagocytized and degraded in the reticuloendothelial system (RES) before reaching the target site [7]. Thus, in order to achieve the long blood circulated half-life, and overcome immune barriers, nanoparticles must be camouflaged with stealth materials [8,9]. Among various types of such materials, Poly(Ethylene Glycol) (PEG) is the most frequently used material to produce stealth nanoparticles [9].

Pluronic F127 belongs to poloxamer group, which is biocompatible and is widely used in clinics for various purposes [10,11]. F127 is an amphiphilic polymer which consists of two PEG chains and one Poly(Propylene Oxide) PPO block. Therefore, F127 has been used as nonionic emulsion surfactant, as well as stealth material in many studies [12,13,14]. In emulsion, the hydrophobic PPO block can anchor into the organic phase while the hydrophilic PEG blocks are exposed to the water phase. After the evaporation of the organic phase, the PPO blocks absorb the nanoparticles hydrophobic surface, whereas the PEG blocks create the new nanoparticle stealth surface [15].

Folic acid is the most frequently employed targeting ligand since its receptor is significantly overexpressed on many types of cancer, whilst it presents in low and non-detectable levels in normal cells [16,17,18]. Thus, folic acid has been conjugated to nanoparticles in order to achieve the active targeting accumulation into the target tumor [18].

In our study, we aim to develop F127-Folate coated SPION as an MRI contrast agent. F127-Folate coated SPION specifically target the folate expressing cancer cells, which can provide the contrast in MRI.

## 2. Materials and Methods

All reagents were purchased from Sigma-Aldrich, St Louis, MO, USA unless otherwise specified.

### 2.1. Conjugation of Pluronic F127 and Folic Acid

**Figure 1:**
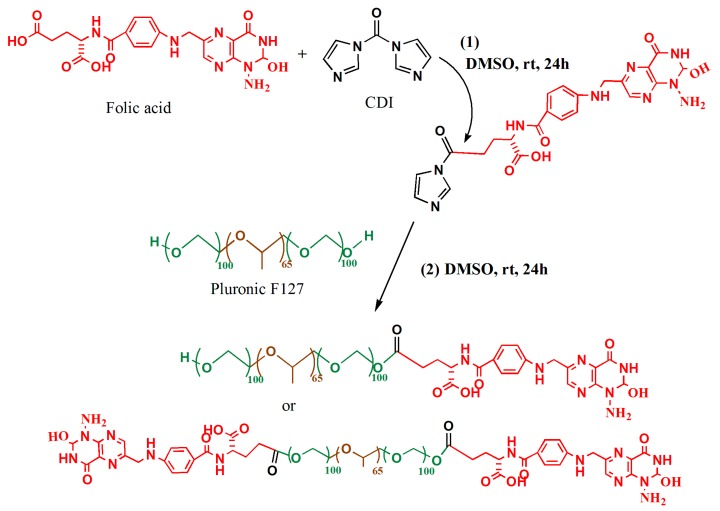
Schematic illustration of chemical reaction. (1) Activation of folic acid by 1,1′ Carbonyldiimidazole (CDI) in dry dimethyl sulfoxide (DMSO) and darkness for 24 h, (2) conjugation of CDI-Folate to Pluronic F127, 24 h darkness.

The conjugation of folic acid to Pluronic F127 was done as described by Jia-Jyun et al., 2009. [19] (Figure 1). Briefly, folic acid (0.4 mmol) was activated by 1,1′ Carbonyldiimidazole (CDI) (0.44 mmol) in 6 mL of dry dimethyl sulfoxide (DMSO) and stirred for 24 h in a dark place. Dry F127 (0.1 mmol) was then added to the mixture and stirred for another 24 h at room temperature. Following this, the reaction was diluted with 50% distilled water and dialyzed (tube: MWCO 3500) against deionized water for 3 days (water was changed twice a day). Next, the solution was freeze-dried for 3 days. The lyophilized outcome was further purified by dissolving in acetone and filtration. The final product was again freeze-dried before being analyzed by NMR and stored at −20 °C until use.

### 2.2. Synthesis Magnetic Nanoparticles

Magnetic nanoparticles SPION were prepared by the co-precipitation of Fe(III) and Fe(II) in an alkaline solution [15]. Briefly, a mixture of 0.003 mole FeCl_3_·6H_2_O, 0.015 mole FeCl_2_·4H_2_O and 500 µL of oleic acid (90%) in 45 mL deionized water was vigorously stirred under nitrogen pressure for 30 min. While being stirred for 30 min, 3 mL of 5 M NH_4_OH was slowly dropped to the mixture. The black slurry product was extensively washed three times with deionized water, re-dispersed in 15 mL n-hexane and sonicated for 10 min. The solution was centrifuged at 12,000 rpm for 20 min, and the supernatant was discarded. The pellet was re-dispersed in 15 mL of *n*-hexane and vigorously vortexed. The solution was again centrifuged at 2000 rpm for 10 min to eliminate the large aggregation.

### 2.3. Iron Determination

The SPION (10 µL) were reduced in 10 µL of HCl 37% before introducing 180 µL of 0.2 X of FerroZine agent (Hatch, Germany). The mixture was incubated for 10 min in a 96-well plate. A standard curve was built in every measurement with three replicate wells. The absorbance of samples was read at 562 nm wavelength by Fluostar Otima (BMG Labtech, Otenberg, Germany).

### 2.4. F127-Folate Coated SPION and F127 Coated SPION Preparation and Particles Characterization

The SPION (5 mg) in 1 mL n-hexan was mixed with 13 mg F127 and 2 mg F127-Folate in 10 mL deionized water. The mixture was emulsified by vortexing for 2 min and further sonicated in water bath for 10 min. The n-hexan was allowed to evaporate overnight by magnetic stirring at 200 rpm/min. The nanoparticles were further washed with deionized water by centrifugation at 30,000 g for 30 min. This process was conducted in triplicate.

For fluorescent labeling, Nile Red (0.05 mg)—a hydrophobic fluorescent dye- was added to the n-hexane before emulsion. Nile Red absorbed to the hydrophobic layer of particles, thus making F127-Folate coated SPION/Nile Red [19].

**Figure A1:**
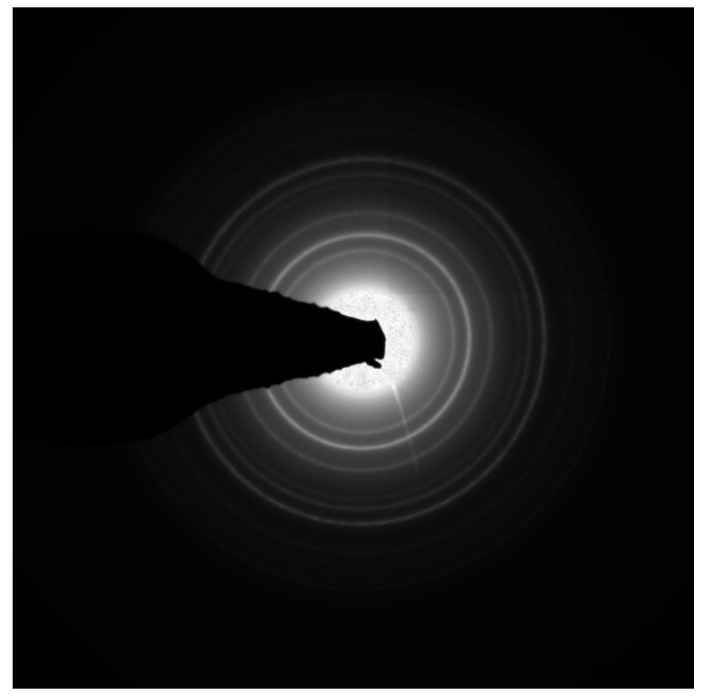
Diffraction of SPION. Magnetite Fe_3_O_4_: (1) 3; (2) 2.56; (3) 2.11; (4) 1.73; (5) 1.63; (6) 1.49; (7) 1.29. Diffraction pattern confirms the main chemical structure of nanoparticle is Fe_3_O_4_.

The particle core size, morphology and diffraction (Figure A1) were also visualized by TEM instrument (TEMEDAX-20, Hillsboro, OR, USA) on a carbon coated copper grid.

The particle hydrodynamic size, polydispersity index (PDI), and zeta potential were characterized using Malvern Zetasizer Nano ZS instrument, Wocestershire, UK. The F127 coated SPION were synthesized in the same way and used as the control nanoparticles. 

### 2.5. Cell Viability and Iron Uptake Concentration

KB cells were seeded at the density of 10,000 cells per well in a 96-well plate and incubated overnight in folic acid free RPMI 1640, 10% Fetal Bovine Serum (FBS), 1% antibiotic mixture (100 u/mL Penicillin, and 100 µg/mL Streptomycin) (Gibco). On the following day, cells were incubated with various concentrations of F127 coated SPION and F127-Folate coated SPION from 100 µg/mL to 3.125 µg/mL for 24 h (four replicate wells). Cell viability was done following the MTT assay (Sigma, St. Louis, MO, USA) while the iron concentration was determined as previously described.

### 2.6. Prussian Blue Staining

The F127–Folate coated SPION and F127 coated SPION (50 µg Fe/mL) were inoculated into 8 well tissue culture chambers (Sarstedt, Germany) and incubated for 3 h. Following this, the cells were washed 3 times with PBS and fixed in HCHO (4%) for 30 min. Iron nanoparticles were stained with a freshly mixed equal volume of HCl (4%) and potassium ferrocyanide (4%) for 15 min. After that, cells were rinsed twice with water and incubated with 250 µL Nuclear Fast Red for 5 min. In the final step, cell chambers were rinsed with water, dried, and mounted with cover slips.

### 2.7. Confocal Laser Scanning Imaging

For confocal laser scanning microscope (CLSM), cells were incubated with F127-Folate coated SPION/Nile Red and F127 coated SPION/Nile Red for 3 h. For the last 15 min of incubation, cell membranes were stained with 50 µg/mL wheat germ agglutinin- Alexa 488 (Life Technology, Carlsbad, CA, USA) for 15 min. Chambers were washed 3 times with PBS and fixed with HCHO 4% in 30 min. Cell chambers were rinsed with water, dried, and mounted with prolong gold antifade mountant with DAPI (Life Tecnology, Carlsbad, CA, USA). Fluorescent imaging was taken by confocal laser scanning microscope (Carl Zeiss, Okberkochen, Germany).

### 2.8. Flow Cytometer Assay (FACs)

F127-Folate coated SPION/Nile Red and F127 coated SPION/Nile Red (50 µg/mL) were inoculated into cell cultured wells and incubated for 3 h in the absence and (50 ng/mL) presence of free folic acid. Cells were then washed and harvested for FACs analysis (Beckman Coutler, Pasadena, CA, USA). Four replicated wells were made in total.

### 2.9. Magnetic Resonance Imaging

All of the MRI experiments were performed in a 16.4 Tesla vertical bore Bruker Avance II spectrometer running Paravision 6.0 (Bruker, Billeria, MA, USA) software. Data were analyzed with MATLAB (Mathworks, Natick, MA, USA).

### 2.10. In Vitro Scanning

The amounts of 4 × 10^5^ cells in the FACs experiment were transferred to glass tubes and fixed in 200 µL of 5% agarose. T2 and T2* maps were generated with the multi slice multi echo (MSME) and multi gradient echo (MGE) sequences, respectively. Echo times were varied to estimate the T2 and T2* parameters (MSME: TE = 5 ms to 80 ms; MGE TE = 2 ms to 36 ms) and the repetition time (TR) was 5 s in each case. The field of view (FOV) was 22 × 22 mm^2^, the matrix size 128 × 128, slice thickness (TH) 0.5 mm, and number of acquisitions (NA) 2.

The R2 relaxation of nanoparticles in the Appendix A was estimated by the MSME sequence.

### 2.11. In Vivo MRI Scanning

Tumors were implanted by subcutaneous injection of 10^6^ KB cells to the left flank of Nude Balb/c female mice (Charles River — 8 weeks old). Tumors were allowed to grow for 2 weeks. All of the mice were fed at the Institute of Biomedicine, Aarhus University following institute standard protocol.

Mice were anesthetized with the mixture of Ketamine/Xylazine/PBS during the scan. Tumor bearing mice were scanned with MRI before and 24 h post nanoparticles administration (100 µg Fe/20 g mouse). T2 weighted images were obtained with MSME: FOV 26 × 26 mm^2^, TR/TE 4000/10.13 ms, NA 2, matrix size 128 × 128, TH 1 mm, number of slices: 5. The signal to noise ratio (SNR) of tumor rim, tumor core, whole tumor, and back muscle were estimated using MATLAB. Since the nanoparticles do not target the back muscle, the SNR of back muscle was chosen for signal intensities comparison. To compare the signal-intensity change in the tumor post injection and pre-injection, the signal ratio between tumor and back muscles was calculated. Then, the reduced signal was calculated as shown in the equation below.

(1) $$\%\;reduced\;signal=\;\frac{post-injection\frac{SNR\;\left(tumor\right)}{SNR\;\left(back\;muscle\right)}}{pre-injection\;\frac{SNR\;\left(tumor\right)}{SNR\;\left(back\;muscle\right)}}\times \;100\%$$

## 3. Results and Discussion

Our study focused on developing folate receptor targeting SPION that could target cancer cells and enhance the contrast in MRI. In order to achieve successful accumulation in the tumor, the SPION must have steric surface to prevent them from systemic clearance and a targeting ligand is needed for tumor penetration. In this study, we prepared F127-Folate coated SPION to target the folate receptor expressing cancer cell. Our in vitro experiments, including FACs, CLSM, Prussian blue staining, and MRI have confirmed the specific targeting of F127-Folate coated SPION. The pilot in vivo MRI showed an enhanced contrast in the tumor of targeted nanoparticles.

### 3.1. Synthesis of Pluronic F127- Folate coated and F127 coated SPION

**Figure 2:**
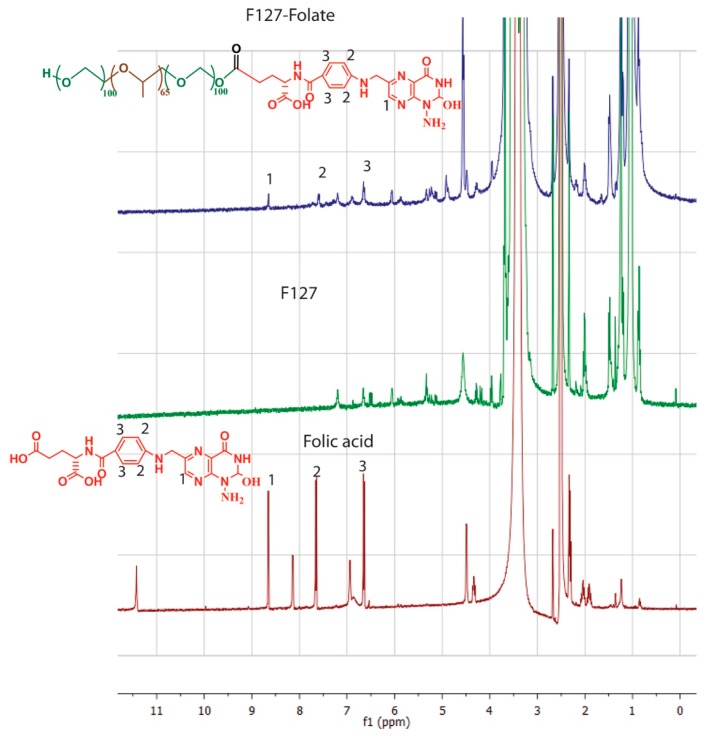
NMR spectrum of (1) folic acid, (2) Pluronic F127, (3) Pluronic F127 – Folate.

The conjugation of folic acid to Pluronic F127 was produced in accordance with the previous report (Figure 1) [19]. Firstly, folic acid was activated by the reaction of its carboxylic group to one of a carbonyldiimidazone group of CDI (reaction 1). Secondly, the remaining carbonyldiimidazone group reacted with the hydroxyl group of F127 (reaction 2). The molar ratio between folic acid: CDI: F127 was 1:1.1:5, so that at least one of the hydroxyl groups of F127 was conjugated to the folic acid. The NMR spectrums of folic acid and F127-Folate (Figure 2) showed the overlay of folic acid signal at peak 1 (8.5 ppm) (pteridine proton), peak 2 (7.6 ppm), and peak 3 (6.6 ppm) (aromatic proton), confirming the success of conjugation.

**Figure 3:**
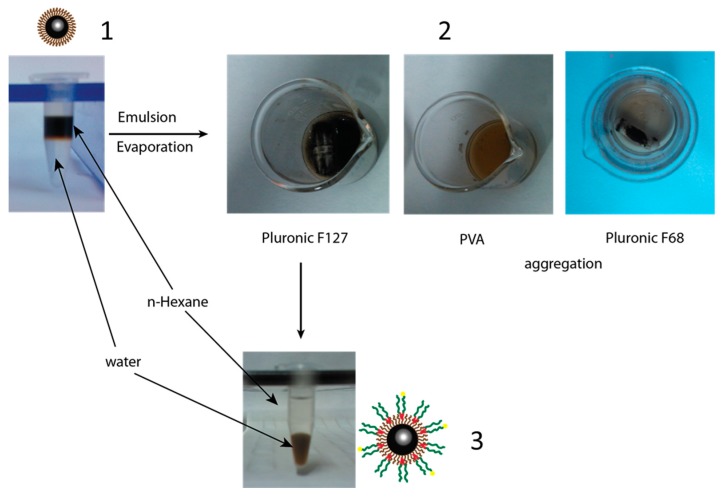
Coating procedure of F127 onto the oleic-super paramagnetic iron oxide nanoparticles (SPION). (1) Oleic SPION dispersed in n-Hexane, (2) polymer coated SPION after the evaporation of *n*-Hexane, (3) the dispersion of F127 coated SPION in water.

The F127 coated SPION were synthesized as shown in Figure 3. In contrast to oleic acid coated SPION, the nanoparticles, after being coated with F127, were well dispersed in water (Figure 3.3). It was hypothesized that the hydrophobic block poly (propylene oxide) of F127 is bound to the hydrophobic oleic layer of SPION, while the hydrophilic block poly (ethylene oxide) exposed itself to the water phase [13,15,20,21]. Besides F127, the same coating procedure was applied to poly (vinyl alcohol) PVA and Pluronic F68 (Figure 3.2). The results showed that SPION were aggregated after the n-hexane evaporation and centrifugation. This result could be due to the non-interaction between hydroxyl group of PVA and oleic acid layers, while the hydrophobic block of F68 was not long enough to anchor onto oleic-SPION surface. Jain et al., (2009) suggest that the ratio between hydrophobic and hydrophilic block of the coating polymer plays an important role during the coating process [13].

### 3.2. Particles Size and Zeta Potential

#### 3.2.1. Size of Particles

**Figure 4:**
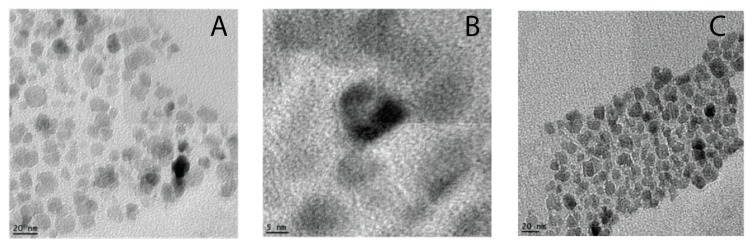
TEM images of SPION. (**a**) Oleic coated SPION, (**b**) oleic coated SPION at magnification 350k ×, (**c**) F127 coated SPION. The scale bars are 20 nm, 5 nm, and 20 nm.

The core size of oleic coated SPION was 12 ± 5 nm (Figure 4a), which was similar to that of the core of nanoparticles after coating with F127 or F127-Folate (Figure 4b). The means that the hydrodynamic size of the nanoparticles was 180 to 190 nm in water, PBS, and cell culture medium, while the PDI of the nanoparticles was mainly below 0.15 (Table 1). Moreover, the nanoparticle sizes in three different environments (water, PBS, culture medium) were stable and well dispersed. Our F127 coated SPION were stable in distilled water for over six months, however nanoparticle size slightly reduced as PDI increased (size/PDI: 185 nm/0.135 versus 156 nm/0.182). It was reported that the stabilization of SPION with F127 at the surface provides the nanoparticles with a steric surface composed of a hydrophilic PEG block. The hydrophilic surface prevents the nanoparticles from aggregating and protein binding [15]. Furthermore, in the systemic condition, the stealth nanoparticles in the size range of 10 to 200 nm could escape from renal filtration and RES [20,22].

#### 3.2.2. Zeta Potential

Nanoparticle charge also has an influence on their blood half-life. Negative charge would reduce the interaction between nanoparticles and blood plasma, thereby enhancing their circulation time [13]. As shown in Table 2, zeta potential of F127 coated SPION and F127-Folate coated SPION were −20.2 mV and −17.15 mV, respectively, which was entirely consistent with previous reports on F127 coated SPION [19,21,23]. These results also supported the explanation about the non-aggregation of nanoparticles in the cell culture medium (Table 1). Thus, F127-Folate coated SPION would have a long circulation time without leading to any aggregation in the bloodstream.

### 3.3. Cell Viability

**Figure 5:**
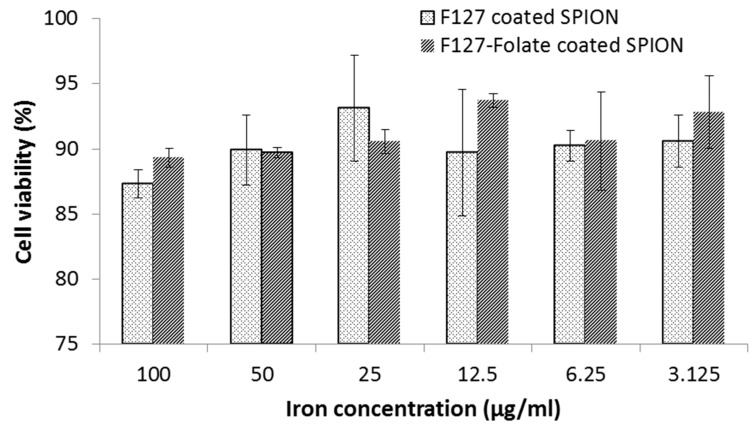
Cell viability of F127 coated SPIO and F127-Folate coated SPION after 24 h of incubation. MTT assay, n = 4, vertical axis: % percentage of viability compares to untreated cells.

Cell viability determines the influence of nanoparticles on cell growth and survival. The results showed that the toxicity slightly rose with the increased concentration of SPION. In comparison between lowest and highest iron concentration used to determine cell viability, the cell viability was 92% and 87% at 3.125 µg/mL and 100 µg/mL, respectively (Figure 5). However, the reduction of cell viability was not statistically significant. Thus, the F127 coated SPION and F127-Folate coated SPION were nontoxic to cells with a long incubation period (24 h). Furthermore, Jia-Jyun et al., (2009) showed that these types of magnetic nanoparticles are safe for the cells, even though a higher iron concentration was used.

### 3.4. In Vitro Uptake of Nanoparticles

Various experiments were carried out in order to prove the enhanced uptake of F127-Folate coated SPION to folate receptor expression KB cells.

#### 3.4.1. Prussian Blue Staining and Confocal Laser Scanning (CLSM)

**Figure 6:**
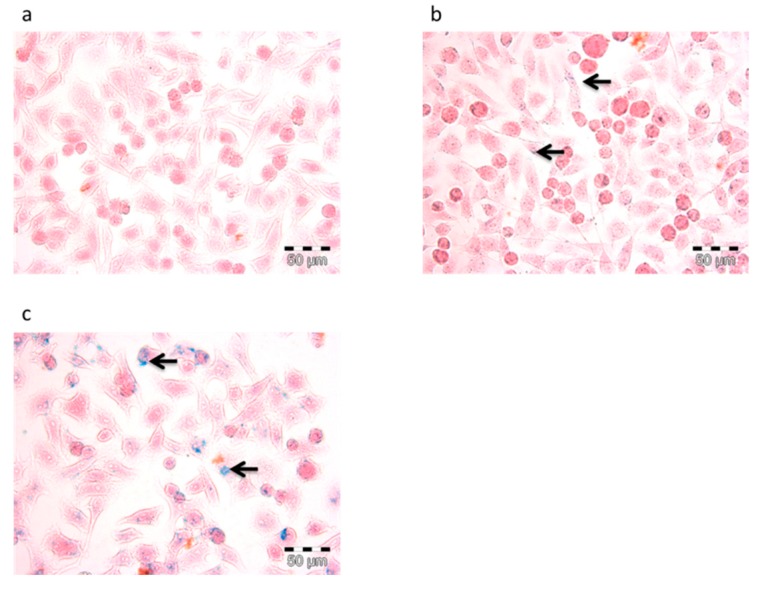
Prussian blue staining of KB cells. Cells were incubated with (**a**) cells only – negative control, F127 coated SPION (**b**), and F127-Folate coated SPION (**c**) for 3 h. Blue color indicates SPION, pink colors indicate cell body, and arrows show the position of iron, scale bar: 50 µL.

**Figure 7:**
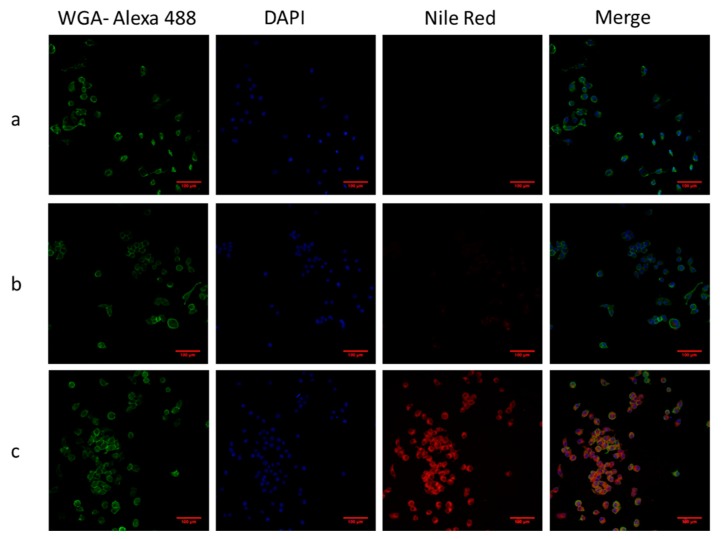
Confocal laser scanning microscope images of particles and KB cells. Cells were incubated with particles for 3 h. (**a**) Negative control, (**b**) F127 coated SPION and Nile Red, (**c**) F127-Folate coated SPION and Nile Red. Green: Wheat germ agglutinin-Alexa fluoro 488, Red: Nile Red, scale bar: 100 µm.

In order to confirm the uptake of nanoparticles in the cells, two staining methods, including Prussian blue and CLSM, were performed. Prussian blue staining is a method to display the accumulation of SPION or iron in cells by forming a complex with potassium ferricyanide. As shown in Figure 6, SPION appeared in blue while cell bodies that were pink. In particular, Figure 6c shows that F127-Folate coated SPION were present inside the cells, while there were only a few of them with the incubation of F127 coated SPION, and none in the negative control. On the other hand, CLSM could determine the uptake of nanoparticles using labeled fluorophores. As shown in Figure 7, the specific targeting of F127-Folate coated SPION/Nile Red to KB cells were visible (Figure 7c), while the control particles had unclear fluorescence signals. Both of the Prussian staining and CLSM results were entirely consistent with each other, indicating the enhanced uptake of F127-Folate coated SPION to KB cells, whereas only an insignificant amount of F127 coated SPION was found in the incubated cells. The results suggest that nanoparticles could accumulate in the cells in both a passive and active way. In the passive way, Pluronic F127 could enable the penetration of nanoparticles through the cell membrane by inserting their hydrophobic chains to the phospholipid bilayers, thereby intensifying an endocytic process [24,25,26]. In the active way, F127-Folate could specifically target and internalize into the cell via folate receptor mediated endocytosis [16,19]. Furthermore, the appearance of iron oxide nanoparticles (Figure 6) and fluorescent dye (Figure 7) in the cells would draw a potential application of multifunctional nanoparticles in molecular imaging and drug delivery [15].

#### 3.4.2. Iron Concentration Measurement and Flow Cytometry Assays (FACs)

**Figure 8:**
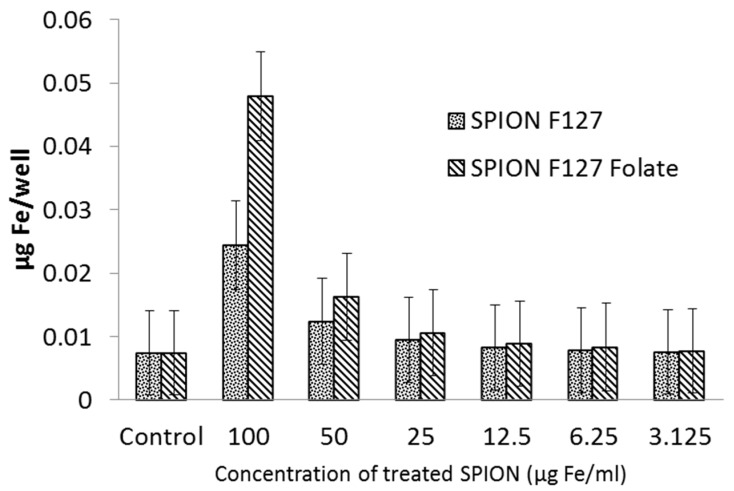
Iron concentration in KB cells, 3 h after incubation. From the left: Cells, 100, 50, 25, 12.5. 6.25, 3.125 µg/mL. (*n* = 4), Y axis: µg/well.

In order to quantify the number of SPION that were taken up by cancer cells, the iron concentration of cells and FACs were confirmed. In the iron concentration measurement, the absorbance of sample after reaction between SPION and Ferrozine were measured. As shown in Figure 8, the iron concentration in cells tended to increase proportionally with incubated nanoparticles concentration. In comparison between two nanoparticle types, there was more iron in F127-Folate coated SPION treated cells, than to the ones treated with F127 coated SPION.

**Figure 9:**
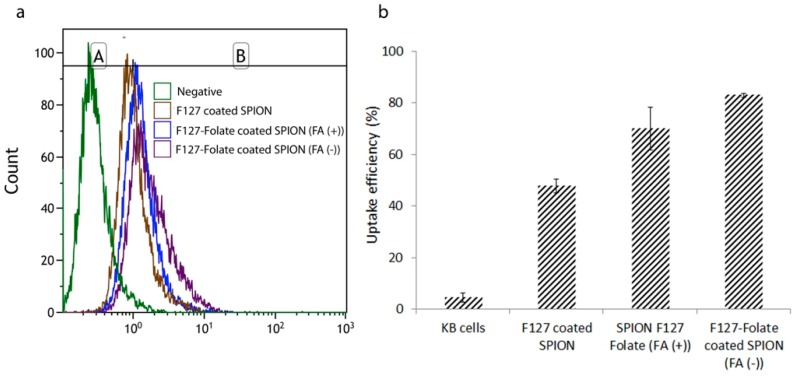
Flow cytometry analysis (FACs). KB cells were incubated with 50 µg/mL iron F127 coated SPION; F127-Folate coated SPION in the media with and without folic acid (5 ng/mL). Histogram (**a**) shows the cell fluorescent signal, graph (**b**) presents uptake efficiency. Gate region A was chosen for non-uptake particles, while gate region B indicates the uptake (n = 4, cell counted number: 10,000, iron concentration for incubation 50 µL/mL).

On the other hand, FACs were used to determine the uptake efficiency of nanoparticles via fluorescent intensities. In the experiment, KB cells were incubated with the nanoparticles in the medium with and without free folic acid (50 ng/mL). As shown in Figure 9, there were more signals in the nanoparticles treated sample than in negative control. More specifically, cells that were incubated with F127-Folate coated SPION showed an uptake enhancement in comparison with cells treated with F127 coated SPION. The results were entirely consistent with the previous iron concentration experiment, where the amount of accumulated F127-Folate coated SPION in the cells was 1.7 times higher than for those were incubated with F127 coated SPION. Furthermore, the F127-Folate coated SPION treated cells in the presence of folic acid, showed lower uptake than that of cells incubated in folic acid free media (65% versus 80%). With the existence of competitive subtracts, folic acid molecules initially target the folate receptor [27] and inhibit the binding of F127-Folate coated SPION; meaning the uptake of nanoparticles was reduced. The result determined the specific folate receptor targeting of F127-Folate coated SPION.

These results are consistent with previous experiments (CLSM and Prussian blue) and other studies. Thereby, it is suggested that our F127-Folate coated SPION could specifically target folate receptor expression cancer cells without causing any harmful consequences [13,19].

### 3.5. Magnetic Resonance Imaging

#### 3.5.1. T2 Relaxation of Nanoparticles

**Figure A2:**
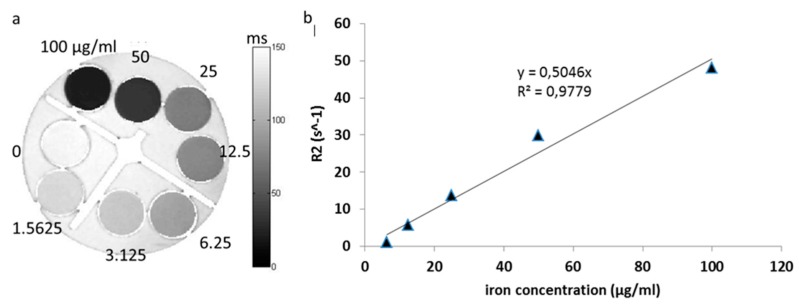
T2 relaxation of F127 coated SPION. F127 coated SPION was diluted in various concentrations, ranging from 100 µg to 1.5625 µg/mL and aliquot to NMR tubes. Next, T2 map was taken by choosing different TE (from 7.76 ms to 62 ms in steps of 10), TR of 5000 ms, number, or average (NA) of 20, matrix size of 256 x 256, field of view (FOV) of 22 × 22 mm^2^, slice thickness (TH) of 0.5 mm. (**a**) T2 map of F127 coated SPION, (**b**) R2 relaxation time graph represents the correlation between iron concentration and R2 relaxation time. Numbers in (**a**) indicate iron concentration (µg/mL).

Figure A2 (Appendix A) shows the exponential curve of R2 relaxation in various iron concentrations. The relaxation between iron concentration and R2 relaxation is a linear curve. In our study, it was a *y* = 0.5046*x*.

#### 3.5.2. In Vitro T2 Relaxation

**Figure 10:**
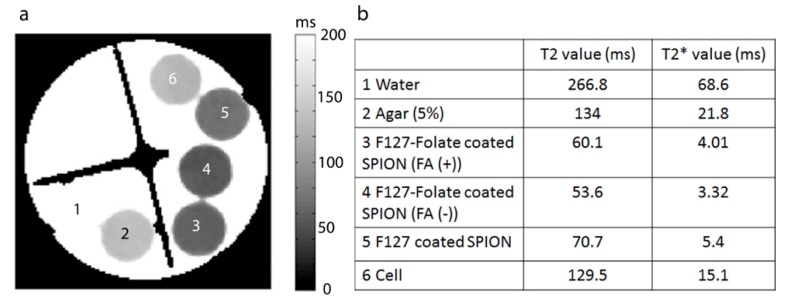
(**a**) T2 map of cells in NMR tubes, (1) water, (2) agarose 0.5%, (3) F127- Folate coated SPION in folic acid media,(4) F127-Folate coated SPION in free Folic acid media, (5) F127 coated SPION, (6) cells in 0.5% agarose. (**b**) T2 and T2* value from T2 map (a) and T2* map (images not show). Number of cells: 4.5 x 10^5^ cells/ 200 µL in 0.5 % agarose.

In order to examine the targeting efficiency and evaluate the MRI contrast agent to the cells, in vitro T2 and T2* relaxation maps were made. As shown in Figure 10, cells that were incubated with SPION showed a reduction in T2 and T2* time compared to negative control (cells and agarose). In particular, F127-Folate coated SPION treated cells showed a shorter T2 and T2* relaxation time compared to non-target F127 coated SPION. Besides, T2 and T2* relaxation times were reduced more considerably in cells that were incubated in the absence of folic acid than cells treated in the presence of folic acid (T2: 53 ms versus 60 ms, T2*: 3.32 ms versus 4.01 ms). This result repeatedly proved the specific targeting and uptake enhancement of F127-Folate coated SPION to folate receptor expressing cells. It also showed great potential for the use of particles for in vivo MRI.

#### 3.5.3. In Vivo T2 Weighted Images

**Figure 11:**
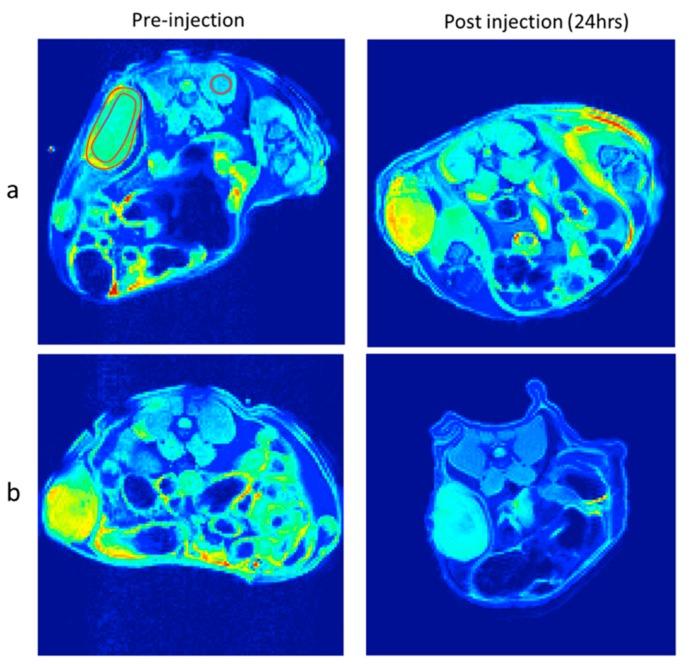
T2 weighted images of tumor bearing mice pre-injection and post injection. F127 coated SPION (**a**) and F127-Folate coated SPION (**b**) nanoparticles. TR/TE 4000/10.16 ms, TH 1 mm, matrix size 128 × 128, number of average: two.

In order to prove the success in cancer targeting in vivo, the nanoparticles should be able to circulate in the system, target cancer via folate receptors, and provide enhanced contrast in MRI. In our study, tumor-bearing mice were intravenously administrated with F127 coated SPION and F127-Folate coated SPION (Figure 11). The signal intensities were measured from different regions including whole tumor, tumor rim, and tumor core. These were compared to back muscle in order to estimate the influence of SPION on T2 weighted MR images after 24 h of injection. The reduced signal from whole tumor, tumor rim, and tumor core relative to back muscle (Table 3) was decreased from about 15% to 20% after F127-Folate coated SPION administration (24 h); meanwhile, no change in the reduced signal in these regions was observed in the mice that were administered with F127 coated SPION. Thus, the reduced signal in the tumor suggests the successful accumulation of F127-Folate coated SPION in the tumor 24 h after injection, while F127 coated SPION were washed out. However, the presence of F127-Folate coated SPION in tumors was random and possibly depended on the distribution of blood vessels [28,29].

This study suggests the potential use of F127-Folate coated SPION as a theranostic agent in the future research. The nanoparticles could specifically provide the enhanced contrast in MRI, and, at the same time, carry chemotherapeutic drugs such as Doxorubicin for chemotherapy.

## 4. Conclusions

In this study, F127-folate coated SPION was synthesized to target folate receptor expressing cancer. After coating, the nanoparticles were stable for a long time and in a number of different environments. In vitro results proved the specific targeting of the nanoparticles to KB cells via folate receptors, as well as the enhanced negative contrast in MRI. The pilot in vivo MRI study showed about 20% CNR reduction in tumor bearing mice that were administrated with F127-folate coated SPION.

## Figures and Tables

**Table 1:** Hydrodynamic size of nanoparticles (DLS) in water, PBS, cell culture medium, and after six months.

Sample	Water–PDI (nm)		PBS–PDI (nm)		Cell Medium–PDI (nm)		Particles after 6 Months, Water (nm)	
F127 Coated SPION	185.2 ± 4.0	0.135 ± 0.01	192.3 ± 9.6	0.138 ± 0.03	182.3 ± 4.2	0.141 ± 0.1	156.7 ± 4.3	0.182 ± 0.02
F127-Folate coated SPION	183.6 ± 2.8	0.122 ± 0.01	194.4 ± 7.8	0.139 ± 0.01	174.4 ± 7.8	0.109 ± 0.02	158.6 ± 4.7	0.175 ± 0.02

**Table 2:** Zeta potential of F127 coated SPION and F127-Folate coated SPION in water. (n = 4).

Sample	Zeta Potential
F127 coated SPION	−20.2 ± 0.5 mV
F127-Folate coated SPION	−17.15 ± 1.8 mV

**Table 3:** The regional signal to noise ratio (SNR) from whole tumor, tumor core, and tumor rim and relatively to SNR from the back muscle.

Position	F127 Coated SPION			F127-Folate Coated SPION		
	Pre-Injection	Post-Injection	Reduced Signal (%)	Pre-Injection	Post-Injection	Reduced Signal (%)
Tumor rim/back muscle	1.5	1.5	99.8	1.6	1.4	85.6
Tumor core/back muscle	1.3	1.5	117.7	1.8	1.4	80.3
Whole tumor/back muscle	1.3	1.4	108.1	1.7	1.4	83.2
